# Dual-specificity tyrosine-phosphorylation regulated kinase 1A Gene Transcription is regulated by Myocyte Enhancer Factor 2D

**DOI:** 10.1038/s41598-017-07655-1

**Published:** 2017-08-03

**Authors:** Pin Wang, Luanluan Wang, Long Chen, Xiulian Sun

**Affiliations:** 1 Otolaryngology Key Lab, Qilu Hospital of Shandong University, No. 107 West Wenhua Road, Jinan, 250012 Shandong Province China; 2 Brain Research Institute, Qilu Hospital of Shandong University, No. 107 West Wenhua Road, Jinan, 250012 Shandong Province China

## Abstract

Dual-specificity tyrosine–phosphorylation regulated kinase 1A (DYRK1A) is localized in the Down syndrome critical region of chromosome 21. As a candidate gene responsible for learning defects associated with Down syndrome and Alzheimer’s disease (AD), DYRK1A has been implied to play pivotal roles in cell proliferation and brain development. MEF2D, a member of the myocyte-specific enhancer factor 2 (MEF2) family of transcription factors, was proved to be in control of neuronal cell differentiation and development. Here we demonstrated that MEF2D could upregulate DYRK1A gene expression through specific activation of DYRK1A isoform 5 gene transcription. A MEF2D responsive element from −268 to −254 bp on promoter region of DYRK1A isoform 5 was identified and confirmed by luciferase assay, electrophoretic mobility shift assay (EMSA) and chromatin immunoprecipitation (ChIP). The coordinated expression of DYRK1A and MEF2D in mouse brain development indicated a possibility of the cross-interaction of these two genes during neurodevelopment. The DYRK1A kinase activity was also affected by MEF2D’s transcriptional regulation of DYRK1A. Therefore, the molecular regulation of DYRK1A by MEF2D further supported their involvement in neurodevelopment.

## Introduction

Down syndrome (DS) is a genetic disorder caused by the presence of all or part of a third copy of chromosome 21^1^. DS is one of the most common chromosome abnormalities in newborns, accounting about 10 per 10,000 live births^2, 3^. DYRK1A, localized in the Down syndrome critical region of chromosome 21, is considered to be a strong candidate gene for intellectual disability related to DS. DYRK1A was first discovered as *minibrain (mnb)* gene and could cause an abnormal spacing of neuroblasts in the outer proliferation center of larval brain in *Drosophila*. These flies exhibit a specific and smaller size of optic lobes and central brain hemispheres. This phenotype indicates a possible function of DYRK1A in regulating neurogenesis^4^. The level of DYRK1A protein gradually decreased with postnatal growth^5^. Several mice models showed that Dyrk1a transgene could lead to neurodevelopmental delay, motor abnormalities, mental retardation, learning and memory deficit and reduced neuronal density^6–8^, which could also be observed in DS patients^9–11^. Overexpression of Dyrk1a might inhibit neural cell proliferation and promote premature neuronal differentiation in the developing cerebral cortex without affecting cell fate and layer positioning^12^, and cause the defects in synaptic vesicle endocytosis as well^13^.

As a transcription factor, MEF2D is initially identified to be critical for muscle cell differentiation^14^. Myocyte-specific enhance factor 2 (MEF2) family members are involved in control of not only muscle but also neuronal cell differentiation and neurodevelopment^15^. Research has revealed that MEF2 was selectively expressed in newly generated postmitotic neurons and was required for the survival of these neurons. Once activated, MEF2 regulated neuronal survival by stimulating MEF2-dependent gene transcription^16^. MEF2 transcription factors are also highly expressed in neurons and are critical determinants of neuronal differentiation and fate^17^. As a member of MEF2 family, MEF2D is concerned to be involved in neurogenesis, neuronal differentiation and survival.

Alternative splicing of DYRK1A mRNA generates more than five transcript variants differing either in the 5′ UTR or in the 3′ coding region^18^. Our recent study showed that DYRK1A isoform 3 gene transcription is regulated by RE1 silencing transcription factor/neuron-restrictive silencer factor (REST) in neurodevelopment in mice and DYRK1A dosage imbalance destabilizes REST protein and reduced its transcriptional activity, thus forming a negative feedback loop in regulation of DYRK1A transcription by REST^19^. We also found that DYRK1A is degraded by E3 ligase SCF^β-TRCP^ and is essential for cell cycle progression^20, 21^. However, the molecular mechanism of DYRK1A transcription during neurodevelopment remained unknown. Here we further elucidate the molecular mechanism of DYRK1A gene transcription. Our data showed that DYRK1A isoform 5 transcription was up-regulated by MEF2D in brain glioblastoma T98G cells. A MEF2D responsive element was identified and functionally analysed in the DYRK1A promoter region between −268 to −254 bp. We also demonstrated that DYRK1A and MEF2D are coordinately expressed in mouse brain during neurodevelopment. The upregulation of DYRK1A by MEF2D increased DYRK1A kinase activity. These results indicated a novel regulatory mechanism for DYRK1A by MEF2D.

## Results

### MEF2D regulates DYRK1A gene transcription

**Figure 1:**
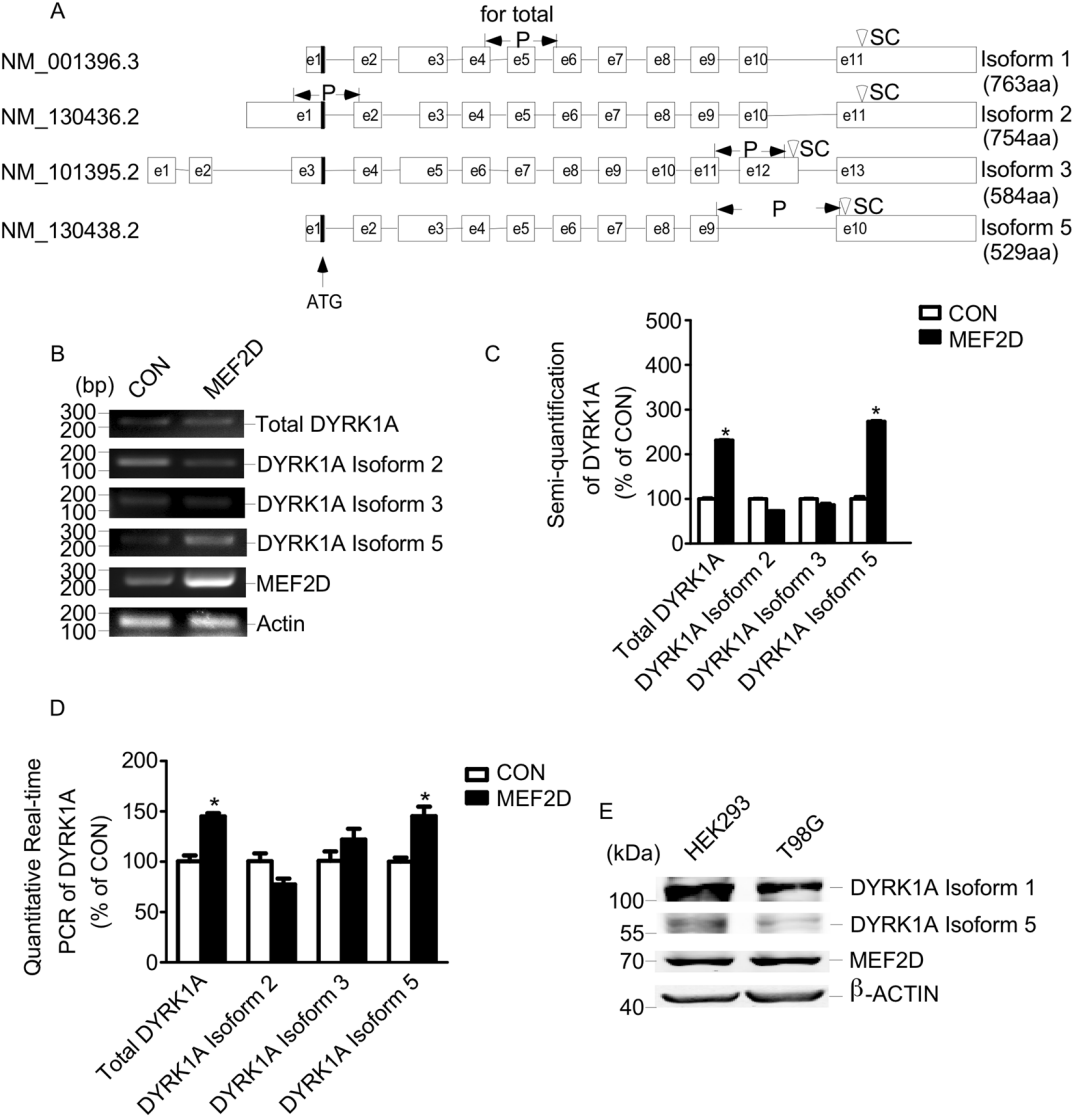
MEF2D regulates DYRK1A gene transcription in T98G cells. (**A**) Genomic organization of DYRK1A isoform 1, 2, 3 and 5 is shown in the scheme. Initiation codon (ATG) was indicated with arrow. E stands for exon. P represents locations of primers for DYRK1A isoforms’ amplification. SC represents stop codons for each isoform. (**B**) MEF2D expression vector and negative control were transfected into T98G cells. mRNA levels of DYRK1A isoforms were determined by RT-PCR. β-actin was amplified as internal control. (**C**) Quantification of **B**. Values represent means ± SEM; n = 3; *P < 0.01 by Student’s t test. (**D**) Quantitative fluorescence real time PCR was performed to detect mRNA levels of DYRK1A isoforms in T98G cells transfected with MEF2D and control vector. Values represent means ± SEM; n = 3; *P < 0.01 by Student’s t test. (**E**) Western blot of DYRK1A isoform 1 and 5 and MEF2D in HEK293 and T98G cells. MEF2D was detected with anti-MEF2D antibody (610774, BD, San Jose, CA, USA). DYRK1A isoform 1 and 5 were detected with anti-DYRK1A antibody (ab156818, Abcam, Shanghai, China). β-ACTIN was used as loading control.

Human DYRK1A gene has nine transcripts that encode for four consensus CDS (ensemble GRCh38.p7). Four consensus CDS of DYRK1A were selected for comparison and analysis (Fig. 1A). DYRK1A isoform 1 (NCBI NM_001396.3) encodes the full length protein while isoform 5 (NCBI NM_130438.2) encodes a shorter protein different in 3′ coding region. We previously cloned and characterized the promoter for DYRK1A isoform 3^19^. The 5′ UTR and exon 1 for isoform 3 and isoform 5 are totally different. Therefore, we propose that there are at least two alternative promoters to regulate the different transcript variants of DYRK1A gene. To examine if MEF2D regulated DYRK1A gene transcription, RT-PCR was performed to analyze the transcription of three isoforms of DYRK1A and total DYRK1A in T98G cells transfected with MEF2D expression vector (Fig. 1B). Total DYRK1A was amplified using a pair of primers spanning from exon 4 to exon 6 of isoform 1 to detect DYRK1A gene expression. The result showed that mRNA expression of total DYRK1A and DYRK1A isoform 5 was significantly elevated with MEF2D overexpression compared with control. MEF2D overexpression increased total DYRK1A expression to 2.3 ± 0.03 folds and DYRK1A isoform 5 expression to 2.7 ± 0.05 folds compared to controls (Fig. 1C). Real time fluorescence PCR also showed that total DYRK1A and DYRK1A isoform 5 mRNA levels were markedly elevated by MEF2D overexpression to 145% ± 11.67 and 145% ± 3.67 of controls (Fig. 1D). Endogenous MEF2D and DYRK1A isoform 5 were detected at protein levels in HEK293 and T98G cells to ensure they are expressed together physiologically (Fig. 1E). These results demonstrated that DYRK1A gene expression could be up-regulated by MEF2D. MEF2D did not regulate the gene expression of DYRK1A isoform 2 and 3, indicating there is an alternative promoter for DYRK1A isoform 5.

### MEF2D specifically activates DYRK1A Isoform 5 promoter

**Figure 2:**
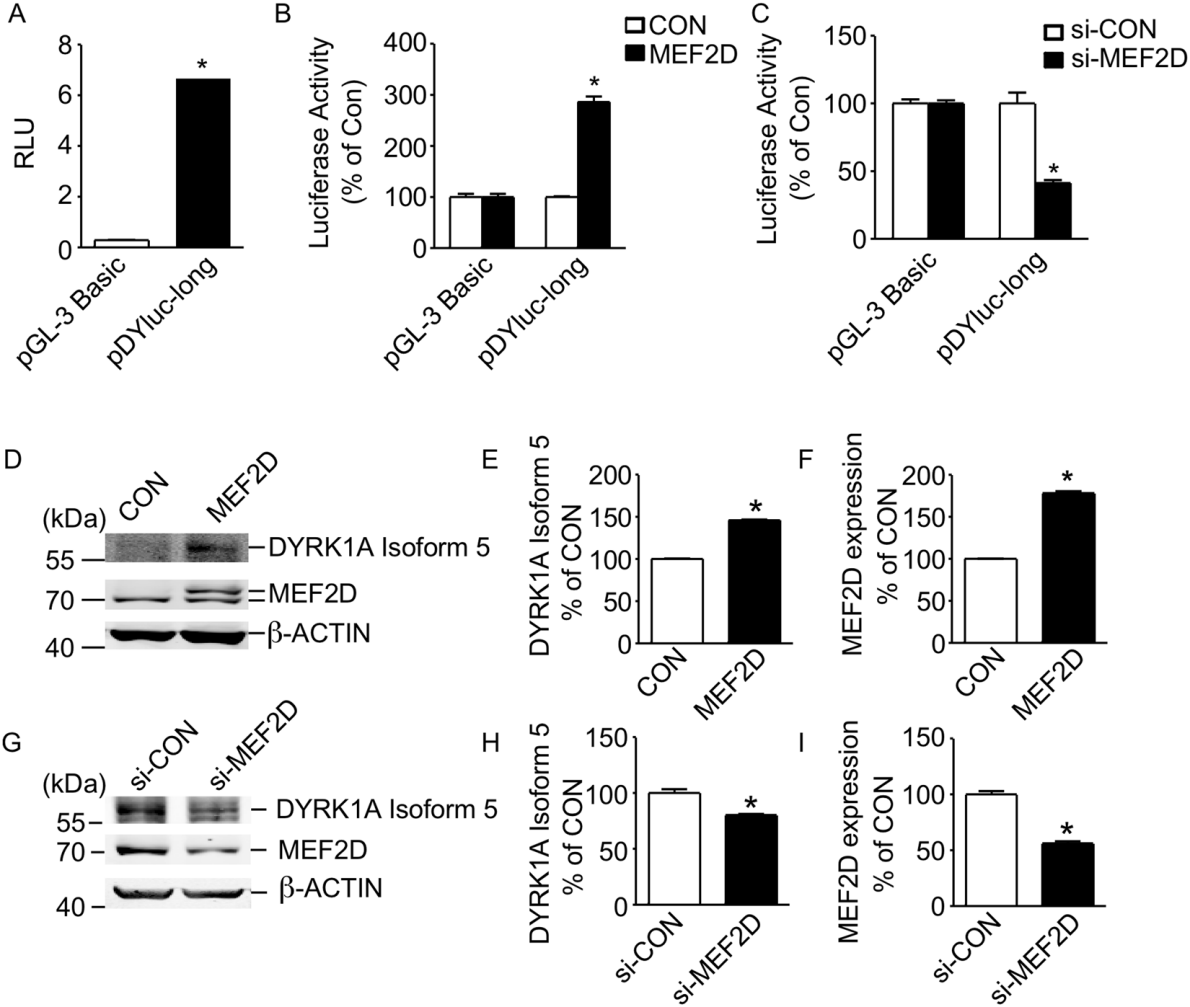
MEF2D specifically activates DYRK1A isoform 5 promoter. (**A**) The DYRK1A promoter construct pDYluc-long, containing the 1825 bp fragment of 5′-UTR from the human DYRK1A isoform 5, was transfected into HEK293 cells. Dual luciferase activity was measured 48h after transfection by a luminometer. The values represent the means ± S.E. (n = 3); *p < 0.01 by Student’s t test. (**B**) MEF2D increases DYRK1A promoter activity. The DYRK1A promoter construct pDYluc-long and pGL-3 basic were co-transfected with MEF2D expression plasmid or empty vector into HEK293 cells. Dual luciferase assay was performed 48 h after transfection. Values represent means ± SEM; n = 3; *P < 0.01 by Student’s t test. (**C**) Knock down of MEF2D leads to decrease of DYRK1A isoform 5 promoter activity compared with control. Dual luciferase assay was performed 48h after transfection. Values represent means ± SEM; n = 3; *P < 0.01 by Student’s t test. (**D**–**F**) Overexpression of MEF2D increased DYRK1A isoform 5 protein level in T98G cells. pCMV6-entry-MEF2D and empty control vector were transfected into T98G cells. Cells were harvested after 48 hours’ transfection. MEF2D was detected with anti-MEF2D antibody (610774, BD, San Jose, CA, USA). DYRK1A isoform 5 was detected with anti-DYRK1A antibody (ab156818, Abcam, Shanghai, China). β-ACTIN was used as loading control. The values represent the means ± SEM; n = 3; *P < 0.01 by Student’s t test. (**G**–**I**) The knockdown of si-MEF2D decreased DYRK1A isoform 5 protein level in T98G cells. T98G cells were co-transfected with si-MEF2D and control. Cells were harvested after 48 hours’ transfection. MEF2D was detected with anti-MEF2D antibody. DYRK1A isoform 5 was detected with anti-DYRK1A antibody (ab156818, Abcam, Shanghai, China). β-ACTIN was used as loading control. The values represent the means ± SEM; n = 3; *P < 0.01 by Student’s t test.

To further investigate the molecular mechanism of DYRK1A gene transcription by MEF2D, an 1826 bp fragment from the 5′ UTR of isoform 5 was amplified by PCR and cloned into pGL3-Basic vector and functionally analyzed. DYRK1A translation initiation point ATG was used as +1 (Fig. 1A). Dual luciferase assay showed this cloned fragment contained significant promoter activity, 6.667 ± 0.1632 RLU compared to 0.2877 ± 0.0304 RLU of pGL3Basic (Fig. 2A). The promoter sequence was analyzed by JASPAR (http://jaspar.genereg.net/). The computer-based transcription factor binding site search revealed that this 1.8-kb region contains 9 predicted MEF2D responsive elements. To test whether the DYRK1A promoter is regulated by MEF2D, DYRK1A promoter construct pDYluc-long was co-transfected with pCMV6-entry-MEF2D expression construct in HEK293 cells. MEF2D expression can be increased by transfection of pCMV6-entry-MEF2D to 1.78 ± 0.02 folds of control (p < 0.0001, Fig. 2D and F). The empty vector was used as negative controls. The results revealed that MEF2D overexpression greatly elevated the activity of DYRK1A promoter to 2.86 ± 0.21 folds of control (Fig. 2B). MEF2D siRNA knockdown was performed using a Stealth RNAi^TM^ siRNA kit (5′-ACUUCCCAGGGAGGCAAAGGGUUAA-3′). MEF2D expression can be decreased by si-MEF2D to 46.53 ± 0.5% of si-con at protein level (Fig. 2G and I). The result revealed that MEF2D knockdown greatly reduced the activity of DYRK1A promoter to 41.36 ± 4.11% of control (Fig. 2C). Meanwhile, MEF2D overexpression increases DYRK1A isoform 5 protein expression to 145 ± 1.5% of control (p < 0.0001, Fig. 2D and E) and knock down of MEF2D by siRNA leads to 20 ± 1% decrease of DYKR1A isoform 5 protein expression (p < 0.0001, Fig. 2G and H). These results exhibited that MEF2D can upregulate DYRK1A gene transcription through the specific activation of DYRK1A isoform 5 promoter and enhance DYRK1A isoform 5 protein expression.

### Identification of MEF2 responsive element on DYRK1A promoter

**Figure 3:**
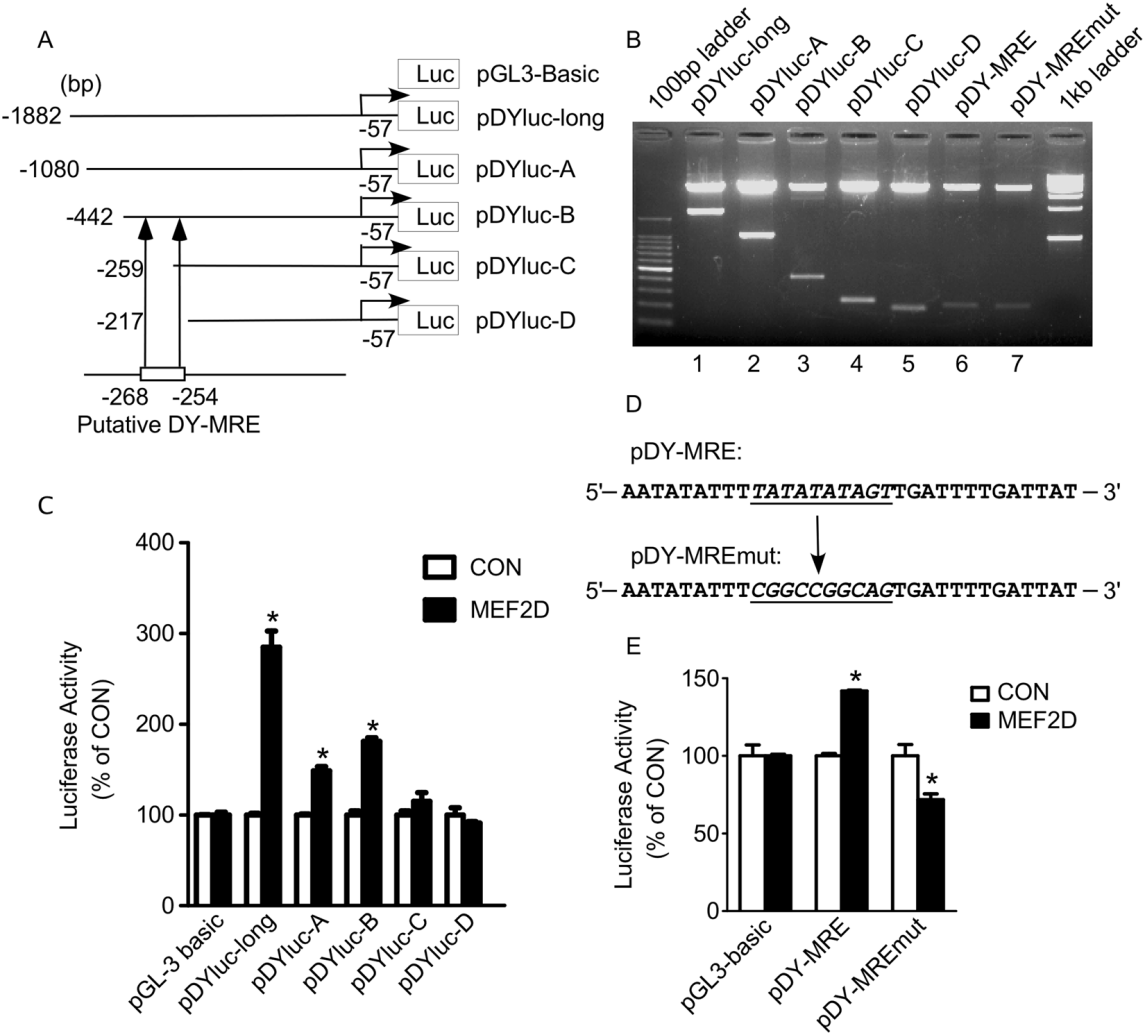
Identification of MEF2 responsive element on DYRK1A promoter. (**A**) Schematic diagrams of the DYRK1A isoform 5 promoter truncation constructs consisting of a 5′-flanking region with serial deletions cloned into the pGL3-Basic plasmid in front of the luciferase reporter gene (Luc). Arrow indicated the direction of transcription. The numbers represent the end points of each construct. +1 is the translation start site. (**B**) The truncation plasmids were confirmed by sequencing and restriction enzyme digestion on a 1.2% agarose gel. Vector size is 4.7 kb, and the DYRK1A gene 5′-flanking fragment inserts ranged from 0.2 to 1.8 kb. (**C**) DYRK1A promoter truncation constructs were transfected into HEK293 cells with MEF2D expression plasmid or empty vector. Dual luciferase assay was performed 48 h after transfection. pGL3-Basic was used as the negative control. Values represent means ± SEM; n = 3; *P < 0.01 by Student’s t test. (**D**) Comparison of mutant sequence and original sequence of putative DY-MRE site at DYRK1A promoter region. (**E**) pDY-MRE and pDY-MREmut were transfected with MEF2D expression vector or empty vector into HEK293 cells. pGL3-basic was used as negative control. Dual luciferase assay was performed 48 h after transfection. Values represent means ± SEM; n = 3; *P < 0.05 by Student’s t test.

Transcription factor MEF2D regulates its target genes through binding to MEF2 responsive elements (MRE). To further examine if DYRK1A promoter contains the MRE, a series of truncation plasmids containing a variety of fragments of DYRK1A isoform 5 promoter region were constructed (Fig. 3A and B). They covered from −1882–57 bp, −1080–57 bp, −442–57 bp, −259–57 bp, −217–57 bp (ATG was used as +1) on the DYRK1A promoter region (lane 1–5 of Fig. 3B). MEF2D expression vector was co-transfected with these truncations plasmids. The empty vector was used as negative control. The luciferase assay indicated that MEF2D significantly increased DYRK1A promoter activity of pDYluc-long (−1882–57 bp), pDYluc-A (−1080–57 bp) and pDYluc-B (−442–57 bp), but had no significant effect on pDYluc-C (−259–57 bp) and pDYluc-D (−219–57 bp) (Fig. 3A and C). This result suggested that the region of −442 bp to −259 bp of DYRK1A promoter contains an effective MRE. Sequence analysis of −442 bp to −259 bp fragment on DYRK1A promoter region showed that there was one putative MRE site located at −268 bp to −254 bp (5′-TTTATATATAGT-3′) (Fig. 3A). To further confirm the putative MRE that was responsible for the MEF2D regulation of DYRK1A, pDY-MRE and pDY-MREmut vectors were constructed containing the putative MRE site and mutant MRE site at DYRK1A promoter region (lane 6 and 7 of Fig. 3B). MRE sequence mutated in pDY-MREmut was shown in Fig. 3D. MEF2D expression vector was co-transfected with pDY-MRE and pDY-MREmut vectors into HEK293 cells. The luciferase assay showed that the MRE mutation in pDY-MREmut abolished the effect of MEF2D on DYRK1A promoter pDY-MRE (Fig. 3E), indicating that the MRE site from −268 bp to −254 bp on DYRK1A promoter was responsive to MEF2D.

### Confirmation of MRE on DYRK1A promoter region

**Figure 4:**
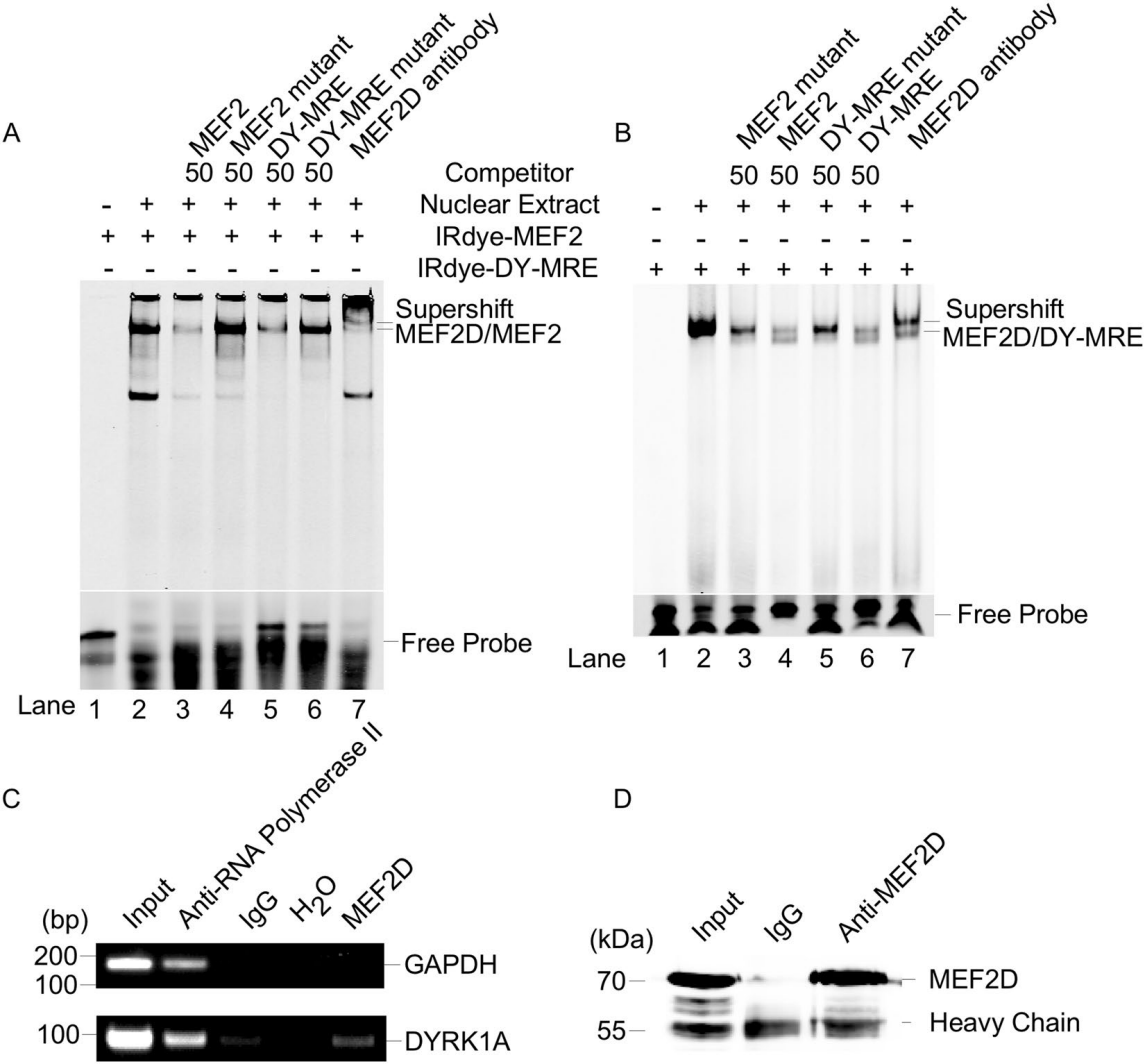
Confirmation of MRE on DYRK1A promoter by EMSA and ChIP. (**A**) The consensus MEF2 oligonucleotides were labelled with infrared fluorescence IRDye® 700 and used as probes. EMSA was performed as described in methods. DY-MRE is the oligonucleotide of −282 bp to −251 bp from DYRK1A promoter. Numbers of competitor indicates the molar excess of labelled oligonucleotides. (**B**) DY-MRE was labelled with infrared fluorescence IRDye® 700 IRDye. EMSA was performed as described in methods. Anti-MEF2D antibody was used in supershift. (**C**) ChIP was used to confirm the binding of MEF2D with DYRK1A promoter region. Anti-MEF2D antibody, anti-RNA Polymerase II and normal mouse IgG were used in chromatin immunoprecipitation from HEK293 cells. Primers to amplify a short DNA sequence spanning the putative DY-MRE site in DYRK1A promoter region and GAPDH were used for PCR. IgG and H_2_O were used as the negative controls. (**D**) Western blot showed that the anti-MEF2D antibody actually immunoprecipitated MEF2D protein. The lower bands were antibody heavy chain.

Electrophoretic mobility shift assay (EMSA) was performed to investigate whether MEF2D binds to this putative MRE from DYRK1A gene promoter. A shifted MEF2D/MRE complex band was detected after incubating the IRDye® 700 labeled consensus MEF2 probe with nuclear extract (Fig. 4A, lane2). Both unlabeled DY-MRE oligoes as well as unlabeled consensus MEF2 oligoes could compete out the shifted MEF2D/MRE complex band with a molar excess of 50 times (lane 3, 5 of Fig. 4A). While mutant DY-MRE oligoes and mutant consensus MEF2 oligoes had no competitive effects (lane 4, 6 of Fig. 4A), suggesting the specificity of the MEF2D/DY-MRE complex. MEF2D antibody could shift the band to a higher molecular weight, further suggesting the specific binding of MEF2D/MRE complex (lane 7 of Fig. 4A). Similar results were obtained when using the IRDye® 700 labeled DY-MRE oligoes corresponding to DYRK1A promoter −268 bp to −254 bp as a probe (Fig. 4B). Addition of nuclear extract shifted the labeled DY-MRE probe to a higher molecular weight band (Fig. 4B, lane2). The cold consensus MRE and DY-MRE oligoes competed out the shifted band (lane 4 and 6 of Fig. 4B), while the mutant consensus MRE or mutant DY-MRE oligoes cannot compete out the shifted band (lane 3 and 5 of Fig. 4B). EMSA showed the MRE site from −268 bp to −254 bp on DYRK1A promoter can bind to MEF2D transcription factor *in vitro*.

Chromatin immunoprecipitation (ChIP) was employed to confirm whether MEF2D binds to the putative MRE site on DYRK1A promoter *in vivo*. Anti-MEF2D antibody was used to immunoprecipitate the MEF2D-DNA in HEK293 cells. Anti-RNA Polymerase II was used as positive control while IgG was used as negative control for ChIP. DY-MRE PCR product was observed in the presence of MEF2D antibody and anti-RNA Polymerase II but not in negative control IgG (Fig. 4C). PCR product of GAPDH observed in the anti-RNA Polymerase II ChIP, but not in the IgG or anti-MEF2D ChIP, was used as PCR control for DY-MRE. The anti-MEF2D antibody actually immunoprecipitated MEF2D protein (Fig. 4D). ChIP-PCR results showed that MEF2D protein effectively immunoprecipitated the genome sequence containing DY-MRE site, indicating that MEF2D binds to the MRE at −268 bp to −254 bp of DYRK1A promoter *in vivo*.

### DYRK1A expression was correlated with MEF2D expression in mice neurodevelopment

**Figure 5:**
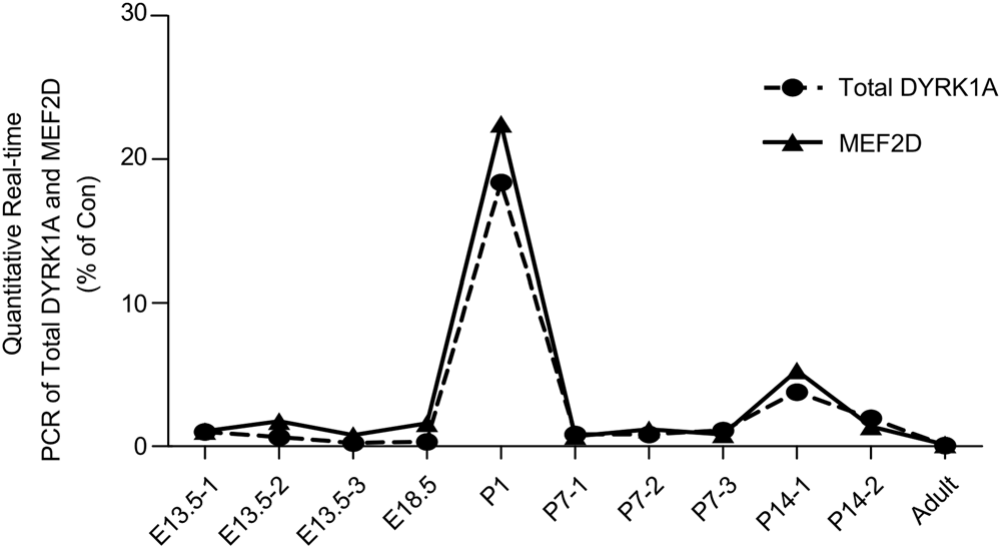
DYRK1A mRNA expression was correlated with MEF2D in mice neurodevelopment. Quantitative real time PCR was performed to detect total DYRK1A and MEF2D mRNA expression. RNA was isolated from normal mouse brain aging at embryonic days 13.5 (E13.5), embryonic days 18.5 (E18.5), postnatal P1, P7, and P14 and adult. One to three mice were used in each time point as indicated by the numbers after the hyphens. p = 0.0478, r = 0.6182 by Spearman correlation test.

DYRK1A expression has been proved to move caudally during embryonic development in chick embryos^22^, suggesting a key role of DYRK1A in embryonic neurogenesis. To further explore the relationship between DYRK1A and MEF2D during neurodevelopment, mRNA expressions were detected with RT-PCR in the developing mice brain. Brains were carefully isolated and RNAs were extracted from normal mice aging at embryonic days 13 and 17, postnatal day 1 (P1), P7, P14 and adult (P30). The quantitative real-time PCR results indicated that mRNA levels of MEF2D and total DYRK1A were coordinately expressed with significant positive correlation during neurodevelopment (p = 0.0478, r = 0.6182 by Spearman correlation; Fig. 5A). These results suggested a possible interaction between DYRK1A and MEF2D in neurodevelopment.

### MEF2D regulates DYRK1A kinase activity exemplified by NFATc2 protein expression

**Figure 6:**
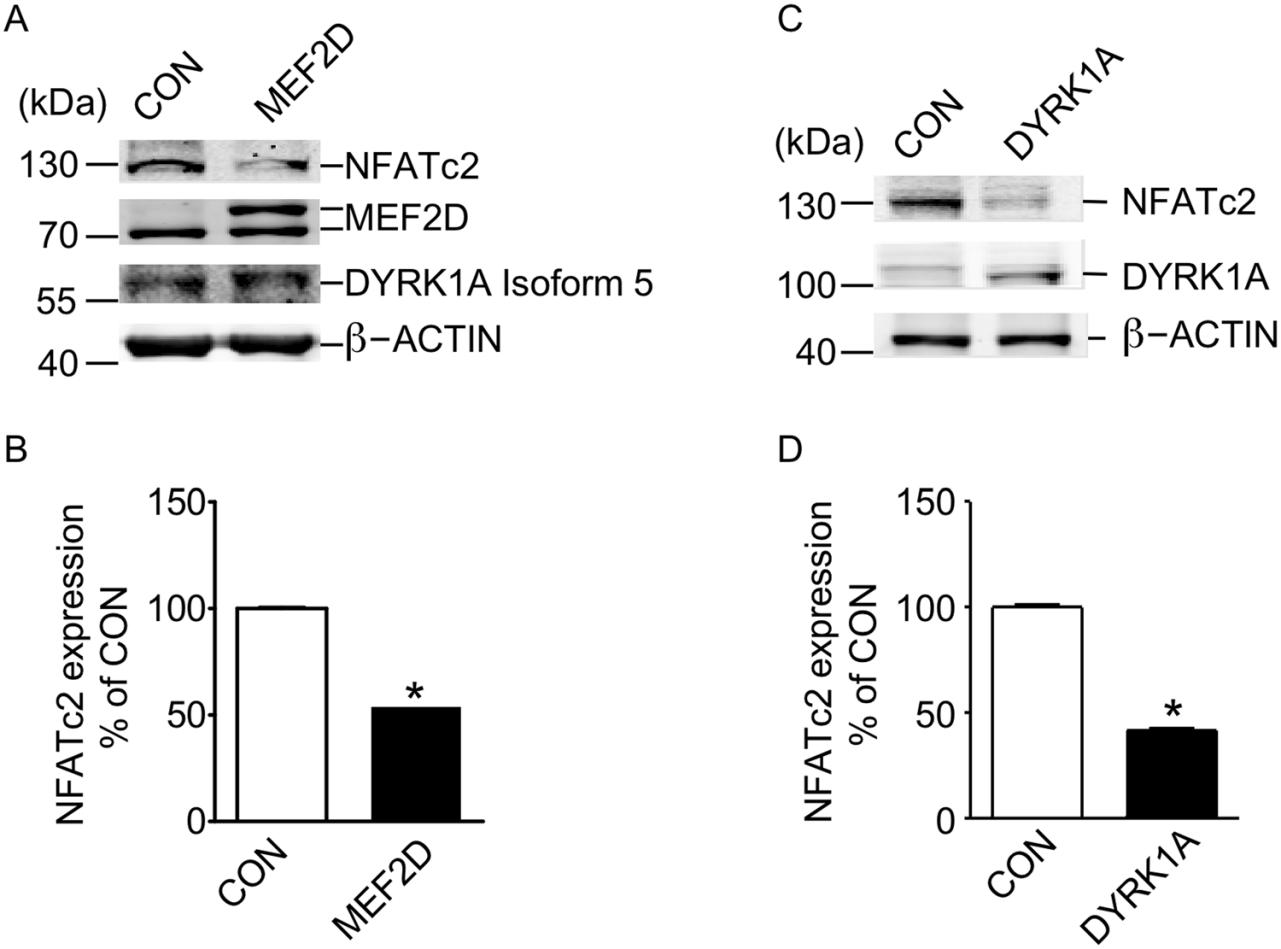
MEF2D regulates DYRK1A kinase activity exemplified by NFATc2 protein expression. (**A**) MEF2D increased DYRK1A protein and decreased NFATc2. MEF2D expression plasmid and empty vector were transfected into T98G cells with NFATc2 expression vector. NFATc2 was detected with anti-HA antibody. MEF2D was detected with anti-MEF2D antibody (610774, BD, San Jose, CA, USA). DYRK1A isoform 5 was detected with anti-DYRK1A antibody (ab156818, Abcam, Shanghai, China). β-actin detected by β-actin monoclonal antibody (SAB1403520; Sigma-Aldrich, Saint Louis, USA) was used as loading control. (**B**) Quantification of A. Values represent means ± SEM; n = 3; *P < 0.01 by Student’s t test. (**C**) DYRK1A decreased NFATc2 protein level. DYRK1A expression plasmid and empty vector were transfected into T98G cells with NFATc2 expression vector. NFATc2 was detected with anti-NFATc2 monoclonal antibody (MA1–025, ThermoFisher, Waltham, USA). DYRK1A was detected with anti-DYRK1A antibody (ab156818, Abcam, Shanghai, China). β-actin detected by β-actin monoclonal antibody (SAB1403520; Sigma-Aldrich, Saint Louis, USA) was used as loading control. (**D**) Quantification of **C**. Values represent means ± SEM; n = 3; *P < 0.01 by Student’s t test.

Previous research has revealed that truncated DYRK1A elevated its kinase activity and increased the ability to phosphorylate its substrate^23^. Transcription factor NFATc2 was a known substrate of DYRK1A and phosphorylation of NFATc2 by DYRK1A decreased NFATc2 protein expression^24^. To investigate if transcriptional regulation of DYRK1A isoform 5 by MEF2D affected DYRK1A kinase activity, T98G cells were co-transfected with MEF2D and NFATc2 expression vectors. We found that MEF2D overexpression markedly decreased NFATc2 expression to 46 ± 0.4% of control while elevated DYRK1A isoform 5 expression to 122 ± 0.1% of control (Fig. 6A) at protein levels (Fig. 6A). DYRK1A overexpression also greatly reduced NFATc2 protein expression to 41 ± 1.5% of control in T98G cells (Fig. 6C and D). These results implied that the DYRK1A kinase activity might be increased by MEF2D (Fig. 6A and B). These results showed that MEF2D not only regulates the DYRK1A gene transcription, but also its kinase activity.

## Discussion

Here our study showed that MEF2D could up-regulate the transcription of total DYRK1A and DYRK1A isoform 5 through binding to a specific DY-MRE at −268 bp to −254 bp at DYRK1A isoform 5 gene promoter region. As a transcription factor, MEF2D binds to sequence-specific DNA and regulates neuronal cell differentiation and development^25–27^. DYRK1A was also proved to play critical roles in regulating cell cycle exit and differentiation^28–31^. Our studies here elucidated the molecular mechanism of DYRK1A isoform 5 gene transcription by MEF2D, further supporting the significant roles of both of them in neurodevelopment.

We previously identified an alternative promoter for DYRK1A isoform 3 that is regulated by REST in neurodevelopment^19^. Compared to the high GC content of isoform 3 promoter, the promoter identified here for isoform 5 did not contain a GC-rich element. Our results also showed distinct regulation of these two alternative promoters as MEF2D specifically regulates isoform 5 gene transcription while it has no effect on isoform 3 expression. Alternative usage of two promoters would contribute to the time- and tissue- specific expression of different DYRK1A variants. The exon 1 for the four isoforms of DYRK1A are all different, implying there may be four alternative promoters for each isoform. Future studies will be needed to characterize the promoters for DYRK1A isoform 1 and 2. There have been few studies on the context-dependent expression of DYRK1A different isoforms. Our and others’ studies showed the dosage of DYRK1A is critical to cellular functions as both overexpression and downexpression may lead to severe diseases, implicating the strict regulation of DYRK1A gene transcription is vital to cell fate and functions. It would be interesting to study the different activation of these two alternative promoters in different tissues and contexts.

DYRK1A has more than five different isoforms that differ with each other from 5′ UTR and 3′ coding region. Although encoding a 234aa shorter isoform which lacks the poly-His domain and having a different C-terminus comparing with the longest isoform 1, DYRK1A isoform 5 may still function similar as DYRK1A isoform 1/2 because it contains both nuclear localization signal and protein kinase domain. Previous research has revealed that Dyrk1a might be proteolyzed into the AD-like truncated forms, which lead to a significant increase of its kinase activity *in vitro* ^23^. They found that the proteolysis of Dyrk1A elevated its kinase activity and increased the ability to phosphorylate Tau in a site-specific manner. According to their results, Dyrk1a truncations molecular weights ranged from 37 kDa to 70 kDa. DYRK1A isoform 5 was 529aa, which might be detected as one of the DYRK1A truncations. It would be interesting to investigate the different kinase activities of different DYRK1A isoforms in the future study.

It was reported that DYRK1A phosphorylated APP at Thr668 and increased Aβ production^32^. DYRK1A also phosphorylated Tau at Thr-212 *in vitro*, a residue that is phosphorylated in filamentous Tau from AD brain^33^. DYRK1A was also proved to phosphorylate Tau at Ser-202 and Ser-404 in both mammalian cells and DYRK1A transgenic mice, leading to disability of Tau to promote microtubule assembly^34^. Dyrk1A overexpression also leads to increase of 3R-tau expression and cognitive deficits in Ts65Dn Down syndrome mice^35^. As DYRK1A is involved in Aβ production and Tau phosphorylation in AD pathology, elevated expression of DYRK1A by MEF2D might contribute to AD pathogenesis^23, 36^.

In early chicken embryos, Dyrk1A is expressed before the onset of neurogenesis in neuroepithelia of the neural tube, neural crest and cranial placodes^22^. Dyrk1A is also proved to be specifically expressed in four sequential developments phases: transient expression in preneurogenic progenitors, cell cycle-regulated expression in neurogenic progenitors, transient expression in recently born neurons, and persistent expression in late differentiating neurons^37^. These results indicate that DYRK1A plays a pivotal role in mouse brain development. We have defined that MEF2D and DYRK1A are coordinately expressed in mouse brain during neurodevelopment. It elucidates a new regulating mechanism of DYRK1A in embryonic brain development and further enriches the evidence of DYRK1A participation in mouse brain neurogenesis.

## Materials and Methods

### Materials

pCMV6-entry-MEF2D construct (RC208748, Origene Co., Beijing, China); pCMV6-entry-DYRK1A construct (RC213183, Origene Co., Beijing, China); NFATc2 expression vector (#11100, Addgene, Cambridge, MA, USA); pGL3-basic vector (E1751, Promega, Madison, WI, USA); Dulbecco’s modified Eagle’s medium (CM15019, MACGENE, Beijing, China); penicillin and streptomycin (C0222, Beyotime); Fetal bovine serum (10099141, ThermoFisher, Waltham, USA); Opti-MEM (51985042, ThermoFisher, Waltham, USA); Lipofectamine^TM^ 2000 transfection reagent (11668019, ThermoFisher, Waltham, USA); Chromatin Immunoprecipitation (ChIP) Assay Kit (17–371, Milllipore, Darmstadt, Germany); Genomic DNA Isolation Kit (DP304–02, TIANGEN, Beijing, China); Dual-Luciferase^®^ Reporter Assay System (E1910, Promega, Wisconsin, USA); Odyssey EMSA Buffer Kit (ABIN2169587, Li-cor, Lincoln, Nebraska, USA); HeLaScribe® Nuclear Extract, Gel Shift Assay Grade (E3521, Promega, Madison, WI, USA); TRI reagent (T9424,Sigma-Aldrich, Saint Louis, USA); SYBR-Green PCR Master Mix (QPK-201,Toyobo, Japan); MEF2D monoclonal antibody (610774, BD, San Jose, CA, USA); NFATc2 monoclonal Antibody (MA1–025, ThermoFisher, Waltham, USA); HA-probe antibody (Y-11, SC-805, santa cruz, California, USA); mouse (C56BL6, Shandong university, china); anti-flag monoclonal antibody (F1804, Sigma-Aldrich, Saint Louis, USA); *β*-actin monoclonal antibody (SAB1403520; Sigma-Aldrich, Saint Louis, USA); anti-DYRK1A antibody (ab156818, Abcam, Shanghai, China); IRDye® 800CW Goat anti-Mouse IgG (925–32210, Licor, Lincoln, Nebraska, USA); IRDye® 680RD Goat anti-Mouse IgG (H + L) (925–68070, Licor, Lincoln, Nebraska, USA).

### Plasmids construction

Genome DNA was isolated from human HEK293 cells. Fragment of 5′ upstream region of DYRK1A isoform 1 was amplified by PCR with primers (5′-CCGCTCGAGGATGATTGGGATGACATCATG-3′ and 5′-CCCAAGCTTCCAGCGGCAAAACTATAAC-3′) and cloned into pGL3-basic vector to make pDYluc-A construct. pDYluc-B construct was obtained from blunt end ligation of products of double digestion of pDYluc-A by EcoRI and XhoI. pDYluc-C and pDYluc-D were obtained by PCR using primers (5′-CCGCTCGAGTAGTTGATTTTGATTATTG-3′, and GLprimer2; 5′-CCGCTCGAGGAATGTTAGAAAATGAA-3′, and GLprimer2) with pDYluc-A as template. The restriction enzymes to generate pDYluc-C and pDYluc-D were XhoI and HindIII. Fragment amplified with primers (5′-CTAGCTAGCGAAACTGGCGAGGTGTAAGTAGCAT-3′, 5′-CCGCTCGAGTCATCCTGCTTAATAATCCTTTTCC-3′) were ligated with insert from pDYluc-A construct. The ligation was double digested by NheI and HindIII and then cloned into pGL-3 basic vector to generate pDYluc-long. Forward primers used for pDY-MRE and pDY-MREmut were 5′-GGGGTACCAATATATTTTATATATAGTTG-3′ and 5′-GGGGTACCAATATATTTCGGCCGGCAGTGATTTTGATTAT-3′ with KpnI as restriction site, respectively. They share the same reverse primer: 5′-CCGCTCGAGTCCAGCGGCAAAACTATAAC-3′ with XhoI as restriction site. They were both cloned into pGL3-basic as well. All the constructed plasmids were confirmed by DNA sequencing. Our deletions of plasmids were sequenced by forward primer RVP3 (5′-CTAGCAAAATAGGCTGTCCC-3′) supported by BioSune Biotechnology Inc. (Jinan, China).

### Cell cultures and transfection

Human HEK293 cells and T98G cells were cultured in Dulbecco’s modified Eagle’s medium containing 10% fatal bovine serum, 4.5 g/L glucose, 1 mM sodium pyruvate, 2 mM L-glutamine, 25 mM HEPES, 100 units/ml penicillin and 0.1 mg/ml streptomycin. Cells were maintained at 37 °C in an incubator containing 5% CO_2_. All of the transfections were carried out with lipofectamine^TM^ 2000 transfection reagent according to the manufacturer’s instruction.

### Real-time quantitative RT-PCR mRNA

All animal protocols were approved by the Animal Care and Use Committee of Shandong University and by the Institutional Ethics Committee on Animal Research of Qilu Hospital, and in compliance with ARRIVE guidelines.

Total RNA was isolated from T98G cells or mouse brain (C57BL/6) by TRIzol reagent. 20–35 cycles of PCR were performed to cover the linear range of the PCR amplification. The mRNA level of DYRK1A gene was quantified using the ABI 7900HT Fast real-time PCR system (Applied Biosystems, Foster City, CA) by SYBR® Green-based gene expression analysis. A comparative CT method (2^−ΔΔCT^) was used to analyse the gene expression level. The primers for real-time quantitative and semi-quantitative PCR were as follows: DYRK1A (221 bp) for all isoforms, forward, 5′-GGATCGTTACGAAATTGACTCCT-3′, and reverse, 5′-ACATAAAGTGGCGTTTCAAATGC-3′; MEF2D (231 bp), forward, 5′-CGTGCTATGTGACTGCGAGAT-3′, and reverse, 5′-CGTGCTATGTGACTGCGAGAT-3′; DYRK1A isoform 2 (157 bp), forward, 5′-CTCAGTTGGGGTAATTGTCTTGC-3′, and reverse, 5′-TCTCTCCTCCTGTATGCATCGTCT-3′; DYRK1A isoform 3 (174 bp), forward, 5′-CAAGCTCAGGTGGCTCATCG-3′, and reverse, 5′-TGGCAGGTGACACAAGCAAA-3′; DYRK1A isoform 5 (221 bp), forward, 5′-GTCAAGCTCAGGTGCGTCAG-3′, and reverse, 5′-CCGGTTACCCAAGGCTTGTT-3′; β-actin (141 bp), forward, 5′-GACAGGATGCAGAAGGAGATTACT-3′; and reverse, 5′-TGATCCACATCTGCTGGAAGGT-3′.

### Immunoprecipitation and Immunoblotting

The cell lysates were resolved by 10% and 12% Glycine SDS-PAGE, and immunoblotting was performed as previously described^38^. The primary antibody used were MEF2D monoclonal antibody, anti-HA polyclonal antibody, DYRK1A polyclonal antibody, and β-actin monoclonal antibody according to the manufacturer’s instruction. Detection and quantification were achieved by the Li-Cor Odyssey imaging system and the software.

### Electrophoretic mobility shift assay (EMSA) and chromatin immunoprecipitation (ChIP)

EMSA and ChIP were performed as described^19^. The association of endogenous MEF2D with DYRK1A promoter in nuclear extraction was confirmed using a chromatin immunoprecipitation assay kit (#17–371, Millipore) following the manufacturer’s protocol The sense sequences of DYRK1A MRE, mutant DYRK1A MRE, consensus MEF2 and mutant MEF2 oligonucleotides were 5′-GACAATTGAATATATTTTATATATAGTTGATT-3′, 5′-GACAATTGAATATATTTCGGCCGTAGTTGATT-3′, 5′-CGGATCGCTCTAAAAATAACCCTGTCG-3′, and 5′-CGGATCGCTCGAAACGCGACCCTGTCG-3′, respectively. DYRK1A MRE (DY-MRE) and consensus MEF2 oligonucleotides were both end-labled with IRDye® 700 (Bioneer) to generate double-stranded probes. ChIP experiments were performed following protocols of the Chromatin Immunoprecipitation (ChIP) Assay Kit. HEK293 cells were cross-linked by formaldehyde (final concentration of 1%) for 10 min at 37 °C, and then washed by cold PBS twice. The cells were lysed by 100μl 1% SDS lysis buffer and sheared by sonication. Proteins and DNA were pulled down with MEF2D monoclonal antibody (610774, BD, San Jose, CA, USA). Anti-RNA Polymerase II was used as a positive control and normal mouse IgG as a negative control. The primers for ChIP-PCR were: 5′-TTTTCCGTGGTAATAGGCA-3′ and 5′-CTTCAATAATCAAAATCAAC-3′. Products of ChIP-PCR were separated on a 2% agarose gel. Immunoprecipitation of ChIP proteins were confirmed by Western blot analysis using anti-MEF2D.

### Dual-luciferase Assay

HEK293 cells were harvested in 48 hours after transfection. Dual luciferase assays were achieved following the protocol supplied by dual luciferase reporter assay kit (Promega, E1910) as previously described^39^.

### Data Analysis

All of the experiments were repeated more than three times. For immunoblotting, immunofluorescence, and quantitative real time RT-PCR, one representative picture is shown; quantifications are from more than three independent experiments. The values represent the means ± S.E. The data were evaluated for statistical significance with analysis of variance or Student’s t test analysis. All the P-values had been shown in figure legends. Differences were classified as significant at P < 0.05.
